# Cardiac Complications and Pertaining Mortality Rate in COVID-19 Patients; a Systematic Review and Meta-Analysis

**DOI:** 10.22037/aaem.v9i1.1071

**Published:** 2021-02-13

**Authors:** Amirmohammad Toloui, Donya Moshrefiaraghi, Arian Madani Neishaboori, Mahmoud Yousefifard, Mohammad Haji Aghajani

**Affiliations:** 1 Physiology Research Center, Iran University of Medical Sciences, Tehran, Iran.; 2 Prevention of Cardiovascular Disease Research Center, Shahid Beheshti University of Medical Sciences, Tehran, Iran.

**Keywords:** COVID-19, Cardiovascular System, Heart Injuries, Hospital Mortality

## Abstract

**Introduction:**

Raising knowledge over cardiac complications and managing them can play a key role in their recovery. In this study, we aim to investigate the evidence regarding the prevalence of cardiac complications and the resulting mortality rate in COVID-19 patients.

**Method:**

Search was conducted in electronic databases of Medline (using PubMed), Embase, Scopus, and Web of Science, in addition to the manual search in preprint databases, and Google and Google scholar search engines, for articles published from 2019 until April 30^th^, 2020. Inclusion criterion was reviewing and reporting cardiac complications in patients with confirmed COVID-19.

**Results:**

The initial search resulted in 853 records, out of which 40 articles were included. Overall analysis showed that the prevalence of acute cardiac injury, heart failure and cardiac arrest were 19.46% (95% CI: 18.23-20.72), 19.07% (95% CI: 15.38-23.04) and 3.44% (95% CI: 3.08-3.82), respectively. Moreover, abnormal serum troponin level was observed in 22.86% (95% CI: 21.19-24.56) of the COVID-19 patients. Further analysis revealed that the overall odds of mortality is 14.24 (95% CI: 8.67-23.38) times higher when patients develop acute cardiac injury. The pooled odds ratio of mortality when the analysis was limited to abnormal serum troponin level was 19.03 (95% CI: 11.85-30.56).

**Conclusion:**

Acute cardiac injury and abnormal serum troponin level were the most prevalent cardiac complications/abnormalities in COVID-19 patients. The importance of cardiac complications is emphasized due to the higher mortality rate among patients with these complications. Thus, troponin screenings and cardiac evaluations are recommended to be performed in routine patient assessments.

## Introduction

Severe acute respiratory syndrome coronavirus 2 (SARS-CoV-2) is a novel coronavirus, which emerged in Wuhan, China in December 2019 and has spread to over 200 countries in the world, ever since (1). Coronavirus disease 2019 (COVID-19) mostly causes lower respiratory tract infection symptoms such as fever, which is its most prevalent symptom, cough, and dyspnea (2). Although people with severe type of the disease form the minority of the patients, it is illustrated that mortality rate is the highest among patients who have comorbidities such as cardiovascular diseases (3).

Angiotensin converting enzyme 2 (ACE2) has been shown to be the receptor of the virus in human cells. Since this enzyme is expressed in many organs such as lungs, heart, kidneys and brain, extrapulmonary manifestations and complications are being studied rigorously, all around the world (4). Cardiac involvement is one of the most common causes of death in COVID-19 patients (5). Cardiovascular complications, whether being chronic or acute, can lead to critically imbalanced homeostasis and could be a serious strike to patient’s recovery from the disease (6). Therefore, raising awareness over cardiac complications caused by the disease and managing them as early as possible can play a key role in recovery of the patients. However, there is no comprehensive evaluation of this issue. Therefore, in this study, we aim to investigate the evidence regarding the prevalence of cardiac complications in COVID-19 patients. Moreover, the risk of mortality after COVID-19 related cardiac involvement has also been assessed.

## Methods

**Study design**

The present systematic review and meta-analysis aims to investigate the evidence regarding cardiac complications in patients, confirmed with COVID-19, and to see whether there is any potential association between the prevalence of cardiac complications and their mortality rate. As a result, PECO in the current study is defined as: P (patients): Patients with confirmed COVID-19 disease, E (exposure): SARS-CoV-2 infection, O (outcome): prevalence of cardiac complications and mortality after COVID-19 related cardiac involvement. As for comparison (C in PECO), mortality rate was compared between COVID-19 patients with cardiac complications and patients without any cardiac involvements.

Search strategy

The searching process was initiated by selecting keywords with the help of experts in the field and screening titles of similar articles. Then, using MeSH and Emtree, equivalent and related synonyms were identified. As a result, a search strategy was constructed based on the instructions of electronic databases of Medline, Embase, Scopus, and Web of Science, and an extensive search was performed in each of the mentioned databases for articles published from the February 1^st^ of 2019 until April 30^th^, 2020. The search strategy in Medline database through PubMed search engine is presented in Appendix 1. In addition, a search was performed in preprint databases, Google, and Google scholar to obtain preprinted and possibly missed manuscripts (gray litterateur search). Moreover, references of the obtained review articles were screened to find additional articles.

**Selection criteria**

The inclusion criteria in the present meta-analysis was reporting cardiac complications and cardiac related mortality rate in patients with confirmed COVID-19. Original observational studies were included. Since most COVID-19 studies had a retrospective nature, both retrospective and prospective studies were included. Prevalence of cardiac complications following COVID-19 and its pertaining mortality rate were extracted from cohort and cross-sectional studies. In addition, we added an extra aim to provide evidence on the relationship between cardiac complication occurrence in COVID-19 patients and their mortality. In this section, in addition to cohort and cross-sectional studies, case-control studies were also included. Furthermore, the exclusion criteria were case report studies, review articles, studies that did not report cardiac involvement and studies whose entire target population was patients with cardiac comorbidities as underlying disease. 

**Data collection**

Two independent reviewers screened titles and abstracts of the gathered articles. Then, full texts of the related articles were obtained and included articles were selected and entered the present systematic review and meta-analysis. Finally, a summary of the included studies was recorded using a checklist, consisting of following variables: first author’s name, publication year, country in which the study was conducted in, number of patients, study design, number of patients in which the cardiac complication was assessed in, mean/median age of the patients, number of males among the patients, type of cardiac complication along with its diagnostic method, number of the deceased in patients presenting with cardiac complication (if reported), and number of the deceased among patients without the cardiac complication of the included articles. If several types of cardiac complications were reported in the studies, the number of each cardiac involvement was recorded separately. Any disagreements within the process were resolved using a third reviewer’s opinion.

**Outcome**

The primary outcome of the present meta-analysis was the prevalence of cardiac complications in COVID-19 patients. Secondary outcome was the risk of mortality after COVID-19 related cardiac involvement. Our screening showed that most studies reported abnormal serum troponin level and acute cardiac injury, separately, as cardiac assessments/complications. Other reported cardiac complications were heart failure, cardiomyopathy, cardiac arrest, myocarditis, pericardial effusion, cardiac insufficiency, and myocardial infarction. 

**Quality assessment**

Two independent reviewers scored the quality of the studies according to National Heart, Lung, and Blood Institute (NHLBI) quality assessment tool (7). This tool contains 14 items on study design, patient selection, sample size justification, analysis, timeframe between exposure and outcome, assessment of different level of exposure, validity and reliability of exposure and outcome assessment, blinding status, missing data management, and considering potential confounders in the analysis. Each reviewer independently assessed the articles and categorized each item as low risk, high risk, unclear risk of bias, cannot determine, or not applicable. Any disagreement was resolved by discussion with a third reviewer.

**Statistical analysis**

All analyses were performed using STATA 14.0 statistical program. Analyses were performed in two parts. Initially, the cardiac complications reported in the articles were categorized into 9 subgroups: acute cardiac injury, abnormal serum troponin level, heart failure, cardiac arrest, myocarditis, cardiac insufficiency, pericardial effusion, myocardial infarction, and cardiomyopathy. The prevalence of each cardiac complication among the COVID-19 patients was calculated with a 95% confidence interval (95% CI) using the “metaprop_one” command performed on the total sample size and the number of patients presenting each complication in the included articles.

In the second part of the analysis, the association between the manifestation of cardiac complications/abnormalities and mortality rate was calculated and presented as an overall odds ratio (OR) with a 95% CI using the “metan” command performed on four groups of data: number of the deceased in patients with a cardiac complication, number of the alive in patients with the cardiac complication, number of the deceased in patients without the cardiac complication, number of the alive in patients without the cardiac complication. I^2^ test was performed to assess heterogeneity, and if the heterogeneity among data was considerable, random effect model was used to calculate 95% CI. Egger’s test was used to evaluate publication bias. Finally, a sensitivity analysis was performed to determine whether the results can be considered robust or not. Therefore, in the sensitivity analyses we excluded possible outlier studies. 

## Results

**Study characteristics**

**Figure 1:**
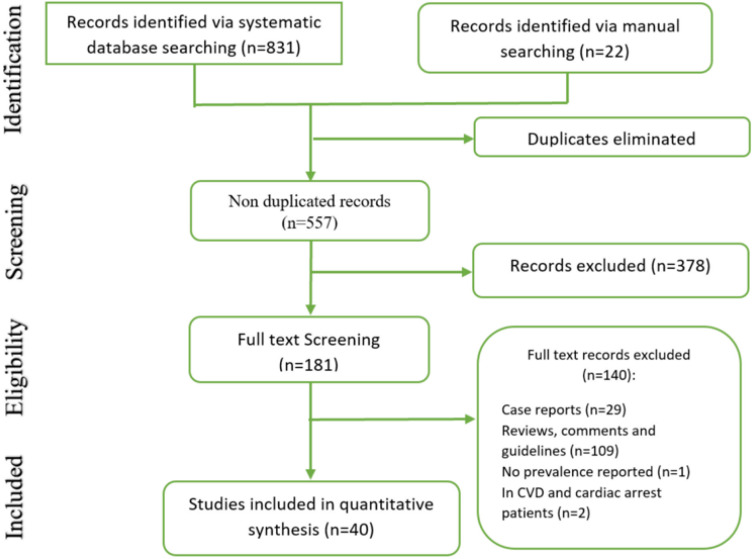
Flow diagram of the present meta-analysis; CVD: Cardiovascular disease

The systematic search resulted in 853 records, and after eliminating duplicates, 557 articles remained. Then, after screening titles and abstracts of the remaining articles, 181 studies were deemed potentially eligible. Afterwards, based on the mentioned exclusion and inclusion criteria, 40 articles were included in the present systematic review and meta-analysis (Figure 1) (5, 8-46). These 40 articles were treated as 60 different experiments, as some studies reported more than one cardiac complication/abnormality. Two studies had taken place in the United States (41, 45), one study was conducted in Spain (9), one study was carried out in Italy (42) and the rest of the included studies were conducted in China. Regarding the study design of the included articles, one study was ambispective (22), four studies were conducted prospectively (9, 14, 19, 39) and the other 35 studies were retrospective. Overall, 15616 patients with confirmed COVID-19 were enrolled in the included studies, with 2985 males among them [patients’ gender was not reported in two of the studies (42, 46)]; however, not all of the patients were assessed for each cardiac complication, so the total number of patients tested for each manifestation is presented for each complication in Table 1. In general, 9 different cardiac complications/abnormalities were reported in the studies including acute cardiac injury, which was reported in 26 studies (5, 8, 11, 13, 16, 18-20, 22-24, 26-30, 32, 33, 35, 37-41, 43, 46), abnormal serum troponin level, reported in 19 studies (8, 10-12, 14, 15, 17, 19, 25, 30, 32, 36-38, 40, 41, 43-45), heart failure, reported in four studies (8, 11, 32, 41), cardiac arrest, reported in three studies (5, 34, 42), myocarditis, reported in two studies (9, 44), cardiac insufficiency, reported in two studies (29, 30), pericardial effusion, reported in two studies (21, 31) and cardiomyopathy (45) and myocardial infarction (32), each reported in one study. Mortality rate was reported in 18 studies (8, 11, 13, 14, 16-18, 20, 26, 29, 30, 33, 34, 37, 39, 40, 44, 46). These numbers were further used to evaluate the odds ratio (OR) between the manifestation of each cardiac complication and the mortality rate in COVID-19 patients. Table 1 demonstrates a summary of the characteristics of the included studies. 

**Risk of bias assessment**

No study had provided a sample size justification, power description, or variance and effect estimates. In addition, 39 studies were not measured to have key potential confounders in their assessment of outcomes. 10 studies did not report the details of their inclusion and exclusion criteria. Moreover, the participation rate of eligible persons was not reported in 6 studies (Table 2).

**Publication bias**

**Figure 2:**
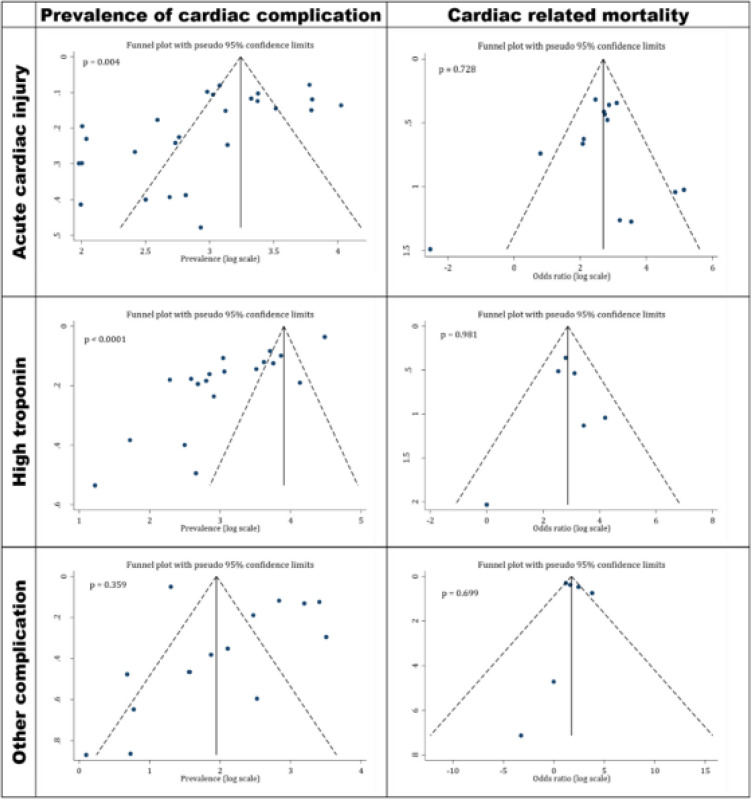
Funnel plot for publication bias in prevalence of cardiac complications in COVID-19 patients and mortality rate after incidence of cardiac manifestation. There are evidences of publication bias among the studies, which reported prevalence of cardiac complications. While, no publication bias was observed regarding the relationship of cardiac complication and mortality in COVID-19 patients

There were evidences of publication bias in the assessment of the prevalence of acute cardiac injury (Coefficient= -3.97; p=0.004) and abnormal serum troponin level (Coefficient = -8.65; p < 0.001) among the included studies. However, no evidence of publication bias was observed in the assessment of cardiac related mortality and the prevalence of other cardiac complications (Figure 2).

**Meta-analysis**

**Figure 3:**
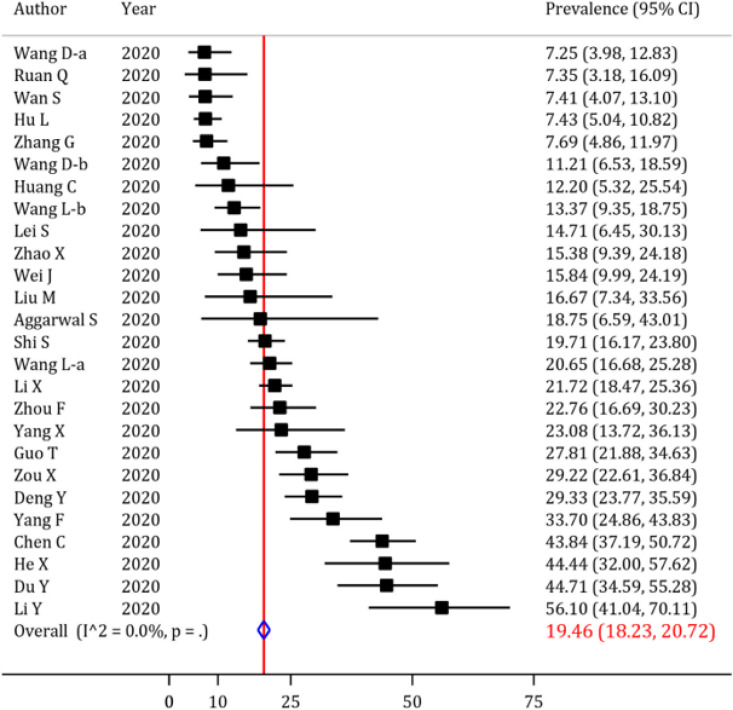
Forest plot for the assessment of the prevalence of acute cardiac injury in COVID-19 patients; CI: confidence interval

**Figure 4:**
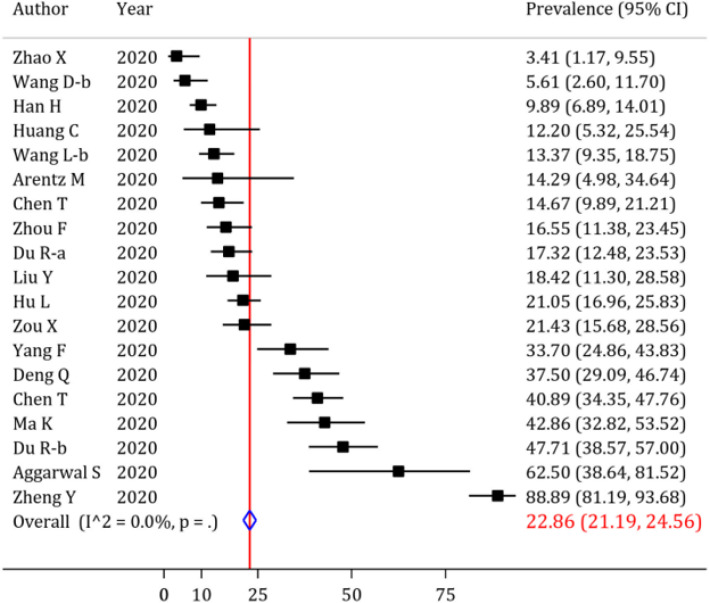
Forest plot for the assessment of the prevalence of abnormal troponin levels in COVID-19 patients; CI: confidence interval

**Figure 5:**
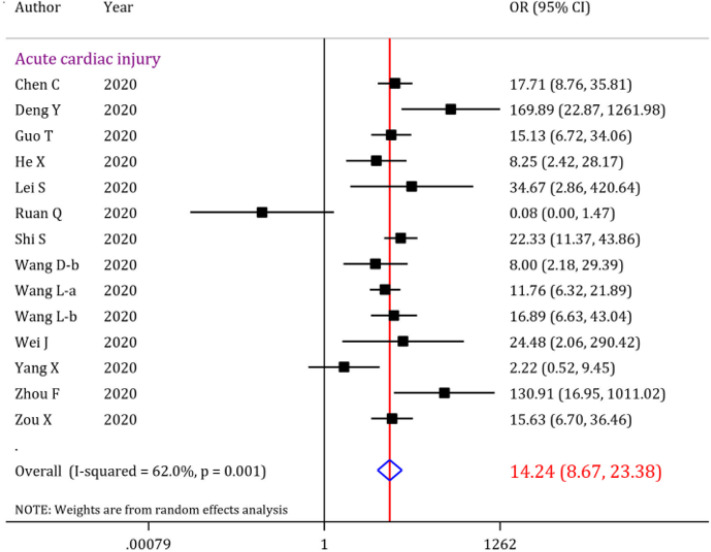
Forrest plot for the assessment of risk of mortality in COVID-19 patients with acute cardiac injury; OR: odds ratio; CI: confidence interval

**Figure 6:**
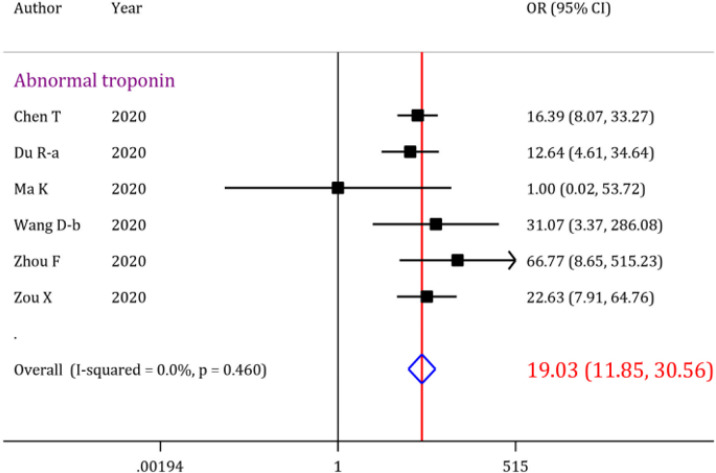
Forrest plot for the assessment of risk of mortality in COVID-19 patients with abnormal troponin level; OR: odds ratio; CI: confidence interval

In the beginning, the prevalence of cardiac complications following SARS-CoV-2 infection was evaluated, and the results are depicted in figures 3, 4, 5 and 6, and table 3. Abnormal serum level of troponin was observed in 22.86% (95% CI: 21.19 to 24.56) of the patients (Figure 4). Moreover, the prevalence of acute cardiac injury, heart failure and cardiac arrest was 19.46% (95% CI: 18.23 to 20.72), 19.07% (95% CI: 15.38 to 23.04) and 3.44% (95% CI: 3.08 to 3.82), respectively (Figure 3 and Table 3). Furthermore, the I^2^ test revealed no heterogeneity regarding the prevalence of abnormal troponin levels and acute cardiac injury. The prevalence of other cardiac complications including myocarditis, cardiac insufficiency, pericardial effusion, myocardial infarction, and cardiomyopathy are depicted in Table 3. Sensitivity analysis showed that after excluding the studies with a high prevalence rate, the prevalence of abnormal serum level of troponin (20.16%; 95% CI: 18.54 to 21.82; cut off for high prevalence rate=50%) and acute cardiac injury (17.17%; 95% CI: 15.94 to 18.43; cut off for high prevalence rate=30) decreased slightly. 

Further analysis revealed that the odds of mortality in COVID-19 patients with acute cardiac injury is 14.24 (OR = 14.24, 95% CI: 8.67 to 23.38) times higher than the COVID-19 patients without acute cardiac injury. However, I^2^ test showed some degrees of heterogeneity regarding the relationship of cardiac complication and mortality of COVID-19 patients (Figure 5). There is a possible outlier study in acute cardiac injury section. After excluding this study, the odds of mortality in COVID-19 patients with acute cardiac injury is increased slightly (OR=15.77; 95% CI: 10.49 to 23.69). In the sensitivity analysis the heterogeneity was decreased (I2=45.5%).

Moreover, the odds of mortality in a COVID-19 patient presenting with abnormal serum troponin level in his/her blood sample was 19.03 (OR=19.03; 95% CI: 11.85 to 30.56) times higher than the patients not having this manifestation. Interestingly, no heterogeneity was observed when calculating the OR for the mortality in COVID-19 patients with abnormal serum troponin level (Figure 6). There are two possible outlier studies in abnormal troponin level section. After excluding these studies, the odds of mortality in COVID-19 patients with acute cardiac injury is increased slightly (OR=17.07; 95% CI: 10.41 to 27.99). The odds of mortality of patients having other cardiac complications are presented in Table 3. 

**Appendix 1:** **Medline search query**

**Search terms**
**“COVID-19 “[Supplementary Concept] OR ”severe acute respiratory syndrome coronavirus 2”[Supplementary Concept] OR ”COVID-19 vaccine”[Supplementary Concept] OR ”COVID-19 diagnostic testing”[Supplementary Concept] OR ”COVID-19 drug treatment”[Supplementary Concept] OR “Betacoronavirus”[mh] OR “Coronavirus Infections”[mh] OR COVID-19[tiab] OR 2019 novel coronavirus disease[tiab] OR COVID19[tiab] OR COVID-19 pandemic[tiab] OR SARS-CoV-2 infection[tiab] OR COVID-19 virus disease[tiab] OR 2019 novel coronavirus infection[tiab] OR 2019-nCoV infection[tiab] OR coronavirus disease 2019[tiab] OR coronavirus disease-19[tiab] OR 2019-nCoV disease[tiab] OR COVID-19 virus infection[tiab] OR Severe Acute Respiratory Syndrom Coronavirus 2[tiab] OR Coronavirus[tiab] OR Coronaviruses[tiab] OR severe acute respiratory syndrome coronavirus 2[tiab] OR 2019-nCoV[tiab] OR Wuhan coronavirus[tiab] OR SARS-CoV-2[tiab] OR 2019 novel coronavirus[tiab] OR COVID-19 virus[tiab] OR coronavirus disease 2019 virus[tiab] OR COVID19 virus[tiab] OR Wuhan seafood market pneumonia virus[tiab] OR Betacoronavirus[tiab] OR Betacoronaviruses[tiab] OR Coronavirus Infections[tiab] OR Coronavirus Infection[tiab] OR Infection, Coronavirus[tiab] OR Infections, Coronavirus[tiab] OR COVID-19 vaccine[tiab] OR COVID-19 diagnostic testing[tiab] OR COVID-19 drug treatment[tiab] OR coronavirus disease 2019 drug treatment[tiab] OR Covid-19 treatment[tiab] OR treatment of Covid-19 virus infection[tiab] OR coronavirus disease-19 drug treatment[tiab] ** **“Troponin”[mh] OR “Heart Injuries”[mh] OR “Myocarditis”[mh] OR “Cardiomyopathies”[mh] OR “Heart Diseases”[mh] OR “Pericarditis”[mh] OR “Cardiovascular Abnormalities”[mh] OR “Cardiovascular Infections”[mh] OR “Cardiovascular Diseases”[mh] OR “Heart Arrest”[mh] OR “Ventricular Dysfunction”[mh] OR “Heart Failure”[mh] OR “Heart Failure, Diastolic”[mh] OR “Heart Failure, Systolic”[mh] OR Heart Injuries[tiab] OR [tiab] OR Injuries, Heart[tiab] OR Heart Injury[tiab] OR Injury, Heart[tiab] OR Myocarditis[tiab] OR Myocarditides[tiab] OR Carditis[tiab] OR Cardiomyopathies[tiab] OR Cardiomyopathy[tiab] OR Myocardial Diseases[tiab] OR Disease, Myocardial[tiab] OR Diseases, Myocardial[tiab] OR Myocardial Disease[tiab] OR Myocardiopathies[tiab] OR Myocardiopathy[tiab] OR Cardiomyopathies, Secondary[tiab] OR Cardiomyopathy, Secondary[tiab] OR Secondary Cardiomyopathies[tiab] OR Secondary Cardiomyopathy[tiab] OR Secondary Myocardial Diseases[tiab] OR Disease, Secondary Myocardial[tiab] OR Diseases, Secondary Myocardial[tiab] OR Myocardial Disease, Secondary[tiab] OR Secondary Myocardial Disease[tiab] OR Myocardial Diseases, Secondary[tiab] OR Heart Diseases[tiab] OR Heart Disease[tiab] OR Cardiac Diseases[tiab] OR Cardiac Disease[tiab] OR Cardiac Disorders[tiab] OR Cardiac Disorder[tiab] OR Heart Disorders[tiab] OR Heart Disorder[tiab] OR Pericarditis[tiab] OR Pleuropericarditis[tiab] OR Endocarditis[tiab] OR Endocarditides[tiab] OR Infective Endocarditis[tiab] OR Endocarditides, Infective[tiab] OR Endocarditis, Infective[tiab] OR Infective Endocarditides[tiab] OR Heart damage[tiab] OR Cardiac injury[tiab] OR Acute cardiac injury[tiab] OR Cardiovascular Abnormalities[tiab] OR Abnormalities, Cardiovascular[tiab] OR Abnormality, Cardiovascular[tiab] OR Cardiovascular Abnormality[tiab] OR Cardiovascular Infections[tiab] OR Cardiovascular Infection[tiab] OR Infection, Cardiovascular[tiab] OR Infections, Cardiovascular[tiab] OR Cardiovascular Diseases[tiab] OR Cardiovascular Disease[tiab] OR Disease, Cardiovascular[tiab] OR Diseases, Cardiovascular[tiab] OR Heart Arrest[tiab] OR Arrest, Heart[tiab] OR Cardiac Arrest[tiab] OR Arrest, Cardiac[tiab] OR Asystole[tiab] OR Asystoles[tiab] OR Cardiopulmonary Arrest[tiab] OR Arrest, Cardiopulmonary[tiab] OR Ventricular Dysfunction[tiab] OR Dysfunction, Ventricular[tiab] OR Dysfunctions, Ventricular[tiab] OR Ventricular Dysfunctions[tiab] OR Heart Failure[tiab] OR Diastolic Heart Failures[tiab] OR Heart Failures, Diastolic[tiab] OR Diastolic Heart Failure[tiab] OR Heart Failure, Diastolic[tiab] OR Cardiac Failure[tiab] OR Heart Decompensation[tiab] OR Decompensation, Heart[tiab] OR Heart Failure, Right-Sided[tiab] OR Heart Failure, Right Sided[tiab] OR Right-Sided Heart Failure[tiab] OR Right Sided Heart Failure[tiab] OR Myocardial Failure[tiab] OR Congestive Heart Failure[tiab] OR Heart Failure, Congestive[tiab] OR Heart Failure, Left-Sided[tiab] OR Heart Failure, Left Sided[tiab] OR Left-Sided Heart Failure[tiab] OR Left Sided Heart Failure[tiab] OR Heart Failure, Systolic[tiab] OR Heart Failures, Systolic[tiab] OR Systolic Heart Failures[tiab] OR Systolic Heart Failure[tiab] OR Troponin[tiab] OR Troponins[tiab] OR Troponin Complex[tiab]** **#1 AND #2**

**Table 1:** Summary of the included studies

Author; Year; Country	Study design	Total study population	Mean age	Number of males	Diagnostic methods	Type of cardiac complication	No. of patients tested for the complication	No. of patients with the complication	No. of deceased in patients with the complication	No. of deceased in patients without the complication
Aggarwal S; 2020; USA	Retrospective	16	67	12	Blood Sample, Echo	Acute cardiac injury	16	3	NR	NR
					NR	Heart failure	16	2	NR	NR
					Blood Sample	Abnormal Troponin	16	10	NR	NR
										
Arentz M; 2020; USA	Retrospective	21	70	11	Blood Sample, Echo, Clinical	Cardiomyopathy	21	7	NR	NR
					Blood Sample	Abnormal Troponin	21	3	NR	NR
										
Baldi E; 2020; Italy	Retrospective	9806	NR	NR	Hospital report	Cardiac arrest	9806	362	NR	NR
										
Barrasa H; 2020; Spain	Prospective	48	63.2	27	NR	Myocarditis	48	1	NR	NR
										
Chen C; 2020; China	Retrospective	150	59	84	Blood Sample	Abnormal Troponin	150	22	NR	NR
										
Chen T; 2020; China	Retrospective	274	62	171	NR	Acute cardiac injury	203	89	72	22
					NR	Heart failure	176	43	41	42
					Blood Sample	Abnormal Troponin	203	83	68	26
										
Deng Q; 2020; China	Retrospective	112	65	57	Blood Sample	Abnormal Troponin	112	42	NR	NR
										
Deng Y; 2020; China	Retrospective	225	54	124	Blood Sample	Acute cardiac injury	225	66	65	44
										
Du Ra; 2020; China	Prospective	179	57.6	97	Blood Sample	Abnormal Troponin	179	31	13	8
										
Du Rb; 2020; China	Retrospective	109	70.7	75	Blood Sample	Abnormal Troponin	109	52	NR	NR
										
Du Y; 2020; China	Retrospective	85	65.8	62	Blood Sample	Acute cardiac injury	85	38	NR	NR
					Clinical	Cardiac arrest	85	7	NR	NR
										
Guo T; 2020; China	Retrospective	187	58.5	91	Blood Sample	Acute cardiac injury	187	52	31	12
										
Han H; 2020; China	Retrospective	273	NR	97	Blood Sample	Abnormal Troponin	273	27	13	8
										
He X; 2020; China	Retrospective	54	68	34	Blood Sample	Acute cardiac injury	54	24	18	8
										
Hu L; 2020; China	Retrospective	323	61	166	Blood Sample	Acute cardiac injury	323	24	NR	NR
					Blood Sample	Abnormal Troponin	323	68	NR	NR
										
Huang C; 2020; China	Prospective	41	49	30	Blood Sample	Abnormal Troponin	41	5	NR	NR
						Acute cardiac injury	41	5	NR	NR
										
Lei S; 2020; China	Retrospective	34	55	14	Blood Sample	Acute cardiac injury	34	5	4	3
										
Li K; 2020; China	Retrospective	83	45.5	44	CT	Pericardial effusion	83	4	NR	NR
										
Li X; 2020; China	Ambispective	548	60	279	Blood Sample	Acute cardiac injury	548	119	NR	NR
										
Li Y; 2020; China	Retrospective	54	61.8	34	Blood Sample	Acute cardiac injury	41	23	NR	NR
										
Liu M; 2020; China	Retrospective	30	35	10	Blood Sample	Acute cardiac injury	30	5	NR	NR
										
Liu Y; 2020; China	Retrospective	76	45	49	Blood Sample	Abnormal Troponin	76	14	NR	NR
										
Ma K; 2020; China	Retrospective	84	48	48	Blood Sample	Abnormal Troponin	84	36	0	0
					Blood Sample, Clinical Symptom	Myocarditis	84	4	0	0
										
Ruan Q; 2020; China	Retrospective	150	NR	NR	NR	Acute cardiac injury	68	5	5	63
										
Shi S; 2020; China	Retrospective	416	64	205	Blood Sample	Acute cardiac injury	416	82	42	15
										
Wan S; 2020; China	Retrospective	135	47	72	Blood Sample	Acute cardiac injury	135	10	NR	NR
										
Wang Da; 2020; China	Retrospective	138	56	75	Blood Sample, ECG, Echo	Acute cardiac injury	138	10	NR	NR
										
Wang Db; 2020; China	Retrospective	107	51	57	Blood Sample	Abnormal Troponin	107	6	5	14
					Blood Sample, ECG, Echo	Acute cardiac injury		12	8	19
										
Wang La; 2020; China	Retrospective	339	69	166	Blood Sample	Acute cardiac injury	339	70	39	26
					Blood Sample, Clinical Symptom	Cardiac insufficiency	339	58	25	52
										
Wang Lb; 2020; China	Retrospective	202	63	88	Blood Sample	Acute cardiac injury	202	27	17	14
					Blood Sample, ECG, Echo	Cardiac insufficiency	202	24	14	19
					Blood Sample	Abnormal Troponin	202	27	NR	NR
										
Wei J; 2020; China	Prospective	101	49	54	Blood Sample	Acute cardiac injury	101	16	3	0
										
Xu X; 2020; China	Retrospective	90	50	39	CT	Pericardial effusion	90	1	NR	NR
										
Yang F; 2020; China	Retrospective	92	69.8	49	Blood Sample	Acute cardiac injury	92	31	NR	NR
					NR	Myocardial infarction	92	6	NR	NR
					NR	Heart failure	92	2	NR	NR
					Blood Sample	Abnormal Troponin	92	31	NR	NR
										
Yang X; 2020; China	Retrospective	52	59.7	35	Blood Sample	Acute cardiac injury	52	12	9	23
										
Yao W; 2020; China	Retrospective	202	63.4	136	Clinical	Cardiac arrest	202	4	0	21
Zhang G; 2020; China	Retrospective	221	55	108	NR	Acute cardiac injury	221	17	NR	NR
										
Zhao X; 2020; China	Retrospective	91	46	49	Blood Sample	Acute cardiac injury	91	14	NR	NR
					Blood Sample	Abnormal Troponin	88	3	NR	NR
										
Zheng Y; 2020; China	Retrospective	99	49.4	51	Blood Sample	Abnormal Troponin	99	88	NR	NR
										
Zhou F; 2020; China	Retrospective	191	56	119	Blood Sample	Abnormal Troponin	145	24	23	31
						Heart failure	145	44	28	26
						Acute cardiac injury	145	33	32	22
										
Zou X; 2020; China	Retrospective	178	60.68	67	Blood Sample, ECG, Echo	Acute cardiac injury	154	45	34	18
					Blood Sample	Abnormal Troponin	154	33	28	24

CT: Computed tomography scan; Echo: Echocardiography; ECG: Electrocardiography; NR: Not reported

**Table 2:** Risk of bias assessment of included studies

**Author; Year**	**Item 1**	**Item 2**	**Item 3**	**Item 4**	**Item 5**	**Item 6**	**Item 7**	**Item 8**	**Item 9**	**Item 10**	**Item 11**	**Item 12**	**Item 13**	**Item 14**
Aggarwal S; 2020	Yes	Yes	No	Yes	No	Yes	Yes	NA	Yes	NA	Yes	NA	Yes	No
Arentz M; 2020	Yes	Yes	Yes	Yes	No	Yes	Yes	NA	Yes	NA	Yes	NA	Yes	No
Baldi E; 2020	Yes	Yes	Yes	No	No	Yes	Yes	NA	Yes	NA	Yes	NA	Yes	No
Barrasa H; 2020	Yes	Yes	Yes	Yes	No	Yes	Yes	NA	Yes	NA	Yes	NA	Yes	No
Chen C; 2020	Yes	Yes	Yes	Yes	No	Yes	Yes	NA	Yes	NA	Yes	NA	Yes	No
Chen T; 2020	Yes	Yes	No	Yes	No	Yes	Yes	NA	Yes	NA	Yes	NA	Yes	No
Deng Q; 2020	Yes	Yes	Yes	Yes	No	Yes	Yes	NA	Yes	NA	Yes	NA	Yes	No
Deng Y; 2020	Yes	Yes	Yes	Yes	No	Yes	Yes	NA	Yes	NA	Yes	NA	Yes	No
Du Ra; 2020	Yes	Yes	Yes	Yes	No	Yes	Yes	NA	Yes	NA	Yes	NA	Yes	No
Du Rb; 2020	Yes	Yes	No	Yes	No	Yes	Yes	NA	Yes	NA	Yes	NA	Yes	No
Du Y; 2020	Yes	Yes	Yes	Yes	No	Yes	Yes	NA	Yes	NA	Yes	NA	Yes	No
Guo T; 2020	Yes	Yes	Yes	Yes	No	Yes	Yes	NA	Yes	NA	Yes	NA	Yes	No
Han H; 2020	Yes	Yes	Yes	Yes	No	Yes	Yes	NA	Yes	NA	Yes	NA	Yes	No
He X; 2020	Yes	Yes	Yes	Yes	No	Yes	Yes	NA	Yes	NA	Yes	NA	Yes	No
Huang C; 2020	Yes	Yes	Yes	Yes	No	Yes	Yes	NA	Yes	NA	Yes	NA	Yes	No
Hu L; 2020	Yes	Yes	Yes	Yes	No	Yes	Yes	NA	No	NA	Yes	NA	Yes	No
Lei S; 2020	Yes	Yes	Yes	Yes	No	Yes	Yes	NA	Yes	NA	Yes	NA	Yes	No
Li K; 2020	Yes	Yes	Yes	Yes	No	Yes	Yes	NA	Yes	NA	Yes	NA	Yes	No
Liu M; 2020	Yes	Yes	Yes	Yes	No	Yes	Yes	NA	Yes	NA	Yes	NA	Yes	No
Liu Y; 2020	Yes	Yes	Yes	Yes	No	Yes	Yes	NA	Yes	NA	Yes	NA	Yes	No
Li X; 2020	Yes	Yes	Yes	Yes	No	Yes	Yes	NA	Yes	NA	Yes	NA	Yes	No
Li Y; 2020	Yes	Yes	Yes	Yes	No	Yes	Yes	NA	Yes	NA	Yes	NA	Yes	No
Ma K; 2020	Yes	Yes	Yes	No	No	Yes	Yes	NA	Yes	NA	Yes	NA	Yes	No
Ruan Q; 2020	Yes	Yes	Yes	No	No	Yes	Yes	NA	Yes	NA	Yes	NA	Yes	No
Shi S; 2020	Yes	Yes	No	Yes	No	Yes	Yes	NA	Yes	NA	Yes	NA	Yes	No
Wang Da; 2020	Yes	Yes	Yes	No	No	Yes	Yes	NA	Yes	NA	Yes	NA	Yes	No
Wang Db; 2020	Yes	Yes	No	No	No	Yes	Yes	NA	Yes	NA	Yes	NA	Yes	No
Wang La; 2020	Yes	Yes	Yes	Yes	No	Yes	Yes	NA	Yes	NA	Yes	NA	Yes	No
Wang Lb; 2020	Yes	Yes	Yes	Yes	No	Yes	No	NA	Yes	NA	Yes	NA	Yes	No
Wan S; 2020	Yes	Yes	Yes	Yes	No	Yes	No	NA	Yes	NA	Yes	NA	Yes	No
Wei J; 2020	Yes	Yes	Yes	Yes	No	Yes	Yes	NA	Yes	NA	Yes	NA	Yes	No
Xu X; 2020	Yes	Yes	Yes	Yes	No	Yes	Yes	NA	Yes	NA	Yes	NA	Yes	No
Yang F; 2020	Yes	Yes	Yes	No	No	Yes	Yes	NA	Yes	NA	Yes	NA	Yes	Yes
Yang X; 2020	Yes	Yes	No	Yes	No	Yes	Yes	NA	Yes	NA	Yes	NA	Yes	No
Yao W; 2020	Yes	Yes	Yes	No	No	Yes	Yes	NA	Yes	NA	Yes	NA	Yes	No
Zhang G; 2020	Yes	Yes	Yes	No	No	Yes	Yes	NA	Yes	NA	Yes	NA	Yes	No
Zhao X; 2020	Yes	Yes	Yes	No	No	Yes	Yes	NA	Yes	NA	Yes	NA	Yes	No
Zheng Y; 2020	Yes	Yes	Yes	No	No	Yes	Yes	NA	Yes	NA	Yes	NA	Yes	No
Zhou F; 2020	Yes	Yes	Yes	Yes	No	Yes	Yes	NA	Yes	NA	Yes	NA	Yes	No
Zou X; 2020	Yes	Yes	Yes	Yes	No	Yes	Yes	NA	Yes	NA	Yes	NA	Yes	No

**NA: Not applicable.**

**Items: **

1. Was the research question or objective in this paper clearly stated?

2. Was the study population clearly specified and defined?

3. Was the participation rate of eligible persons at least 50%?

4. Were all the subjects selected or recruited from the same or similar populations (including the same time period)? Were inclusion and exclusion criteria for being in the study prespecified and applied uniformly to all participants?

5. Was a sample size justification, power description, or variance and effect estimates provided?

6. For the analyses in this paper, were the exposure(s) of interest measured prior to the outcome(s) being measured?

7. Was the timeframe sufficient so that one could reasonably expect to see an association between exposure and outcome if it existed?

8. For exposures that can vary in amount or level, did the study examine different levels of the exposure as related to the outcome (e.g., categories of exposure, or exposure measured as continuous variable)?

9. Were the exposure measures (independent variables) clearly defined, valid, reliable, and implemented consistently across all study participants?

10. Was the exposure(s) assessed more than once over time?

11. Were the outcome measures (dependent variables) clearly defined, valid, reliable, and implemented consistently across all study participants?

12. Were the outcome assessors blinded to the exposure status of participants?

13. Was loss to follow-up after baseline 20% or less?

14. Were key potential confounding variables measured and adjusted statistically for their impact on the relationship between exposure(s) and outcome(s)?

**Table 3:** Summary of findings regarding cardiac complications in COVID-19

**Complication**	**Number of studies**	**Prevalence**	**95% CI**		**Number of studies**	**Odds ratio**	**95% CI**	**P**
Acute cardiac injury	26	19.46	18.23, 20.72		14	14.24	8.67, 23.38	<0.001
Abnormal troponin	19	22.86	21.19, 24.56		6	19.03	11.85, 30.56	<0.001
Heart failure	4	19.07	15.38, 23.04		2	10.66	5.69, 19.97	<0.001
Cardiac arrest	3	3.44	3.08, 3.82		1	0.04	0.00, >999.0	0.651
Myocarditis	2	3.66	0.88, 7.82		1	1.00	0.00, >999.0	>0.99
Pericardial effusion	2	2.62	0.58, 5.73		---	---	---	---
Cardiac insufficiency	2	15.06	12.15, 18.22		2	4.65	2.82, 7.66	<0.001
Cardiomyopathy	1	33.33	17.19, 54.63		---	---	---	---
Myocardial infarction	1	6.52	3.02, 13.51		---	---	---	---

---: No data; CI: Confidence interval

## Discussion

In this systematic review and meta-analysis, we investigated the prevalence of 9 cardiac complications in COVID-19 patients and their subsequent mortality rates. Abnormal serum level of troponin with 22.86% prevalence, detected by laboratory tests, and acute cardiac injury with 19.46% prevalence, diagnosed with laboratory test results and other diagnostic techniques, were among the most prevalent complications/abnormalities observed in COVID-19 patients. Although a large number of studies reported the prevalence of the two mentioned cardiac complications, no heterogeneity was observed in this section. To contemplate even more on the matter, the prevalence of abnormal troponin level and acute cardiac injury are rather close numbers, which might mean that by close observation of troponin changes in a COVID-19 patient, we could possibly anticipate acute cardiac injuries in them and take appropriate measures. However, serum troponin levels also increase due to damage to other tissues, such as the kidneys, which may make it impossible to use troponin level alone to detect cardiac damage. Other cardiac complications such as heart failure and cardiomyopathy were also prevalent among the patients. However, with the small number of the studies observing them, more data is needed on the matter. 

Regarding the limitations, each individual article used a different reference range for troponin or other injury indicators to be classified as abnormal, and each one used different diagnostic and laboratory test results to define acute cardiac injury, which could cause slight differences in the reported prevalence of acute cardiac injury and abnormal serum level of troponin. For example, Huang et al. defined acute cardiac injury as cardiac troponin rising to 3 or more times than normal or appearance of new abnormalities in echocardiography or ECG (19); While many others defined it only with the appearance of abnormal troponin levels in blood samples. Moreover, some articles’ study population only consisted of critically ill patients or deceased ones, which could shift prevalence statistics, causing inevitable heterogeneity in reported numbers (5, 15, 32). Additionally, it is worth mentioning that the mechanism of myocardial injury, whether being done by direct viral invasion to the host tissue or due to imbalanced homeostasis, was of no concern for the writers at the time. Although more research should be conducted to identify the exact causes of this damage, the effects it carries are the most important subject that should be studied at these critical times. Even though limitations were present in data reporting the prevalence of cardiac outcomes, it is important to pay enough attention to these complications, since many of them are directly related to patients’ general situation and illness severity.

On the other hand, our study shows intriguing data regarding mortality rate in patients presenting with cardiac complications, especially acute cardiac injury and abnormal serum level of troponin. Abnormal troponin levels are associated with about 19 times higher mortality risk in COVID-19 patients, which is of great importance in disease management for health care providers around the world. Considering the fact that no heterogeneity was observed regarding the risk of mortality in patients with abnormal troponin level, laboratory screening during routine patient assessments can be critical in early detection of cardiac injury and intervening accordingly. In addition, we suggest that blood levels of troponin should be evaluated as a prognostic factor in patients with cardiac involvement. 

Furthermore, our data indicated that acute cardiac injury could raise the mortality to about 14 times more in COVID-19 patients. However, this number was associated with an overall heterogeneity, which could be attributed to different definitions of cardiac injury in studies, discussed above. Nonetheless, although one can conclude that there is a relationship between acute cardiac injury following COVID-19 and increased risk of mortality, the threat that acute cardiac injury poses to patients’ health status in the future is undeniable and demands careful monitoring and managements when confronting this situation. Moreover, it is noteworthy that articles reporting mortality rates of cardiac complications were mostly focused on abnormal troponin level and myocardial injury and the number of the articles reporting correlations between other cardiac complications such as heart failure, and mortality rate was very few; so, we cannot present an exact estimation of the contribution of other cardiac outcomes such as heart failure and myocarditis to patients’ fatality. In addition, inability to blind the outcome assessors in the studies and some articles not explicitly presenting inclusion and exclusion criteria were further limitations detected in the studies.

Finally, we performed a sensitivity analysis and excluded possible outlier studies. The overall effect size slightly changed and therefore, the results seem to be robust. 

To conclude, our findings thoroughly approve of other published articles investigating associations between COVID-19 cardiac outcomes and related mortalities (47, 48), confirming that cardiac injury, regardless of the mechanism of establishment, is a factor determining disease severity and patient prognosis reliably in large scale and should be considered and monitored from early stages of disease.

## Conclusion

Cardiac complications/abnormalities can be prevalent in COVID-19 patients in the forms of acute cardiac injury, serum troponin levels abnormalities, heart failure, cardiac arrest, and other types. The importance of cardiac involvements is further highlighted when observing the higher mortality rate among COVID-19 patients presenting with cardiac involvements. Thus, careful monitoring of heart involvements should be performed in COVID-19 patients.

## Abbreviations

SARS-CoV-2: Severe Acute Respiratory Syndrome Coronavirus 2

COVID-19: Coronavirus disease 2019

ACE2: Angiotensin Converting Enzyme 2

PECO: Problem, Exposure, Comparison, Outcome

NHLBI: National Heart, Lung, and Blood Institute

CI: Confidence Interval

OR: Odds Ratio

ECG: Electrocardiography

## Ethics approval and consent to participate

This study received ethics approval from Ethics committee of Iran University of medical Sciences.

## Consent for publication

Not applicable.

## Availability of data and materials

All data generated or analyzed during this study are included in this published article [and its supplementary information files].

## Conflict of Interest

There is no conflict of interest.

## Funding

This study was supported by Prevention of Cardiovascular Disease Research Center, Shahid Beheshti University of Medical Sciences, Tehran, Iran.

## Authors’ contribution

Study design: MY, MHA; Data gathering: AT, DM, AMN, MY; Analysis: MY; Interpreting the results: MY, MHA; Drafting: AT, DM, AMN; Critically revised the paper: All authors.
